# Effect of ginsenoside Rh2 on the migratory ability of HepG2 liver carcinoma cells: Recruiting histone deacetylase and inhibiting activator protein 1 transcription factors

**DOI:** 10.3892/mmr.2014.2392

**Published:** 2014-07-18

**Authors:** QINGQIANG SHI, JING LI, ZIQIANG FENG, LVCUI ZHAO, LIAN LUO, ZHIMEI YOU, DANYANG LI, JING XIA, GUOWEI ZUO, DILONG CHEN

**Affiliations:** 1 Laboratory of Stem Cell and Tissue Engineering, Department of Histology and Embryology, Chongqing Medical University, Chongqing 40016, P.R. China; 2 Laboratory of Clinical Diagnostics, Chongqing Medical University, Chongqing 40016, P.R. China

**Keywords:** HepG2, ginsenoside Rh2, activator protein 1, matrix metalloproteinase 3, histone deacetylase

## Abstract

In previous experiments, ginsenoside Rh2 induced apoptosis and cell cycle arrest, which indicates a potential role for ginsenoside Rh2 in anticancer treatment. The effect of ginsenoside Rh2 on cancer is marked and ginsenoside Rh2 has been shown to inhibit pancreatic tumor migratory ability. In the present study, Transwell chambers were used in order to investigate whether ginsenoside Rh2 inhibits the migratory ability of HepG2 liver carcinoma cells. Furthermore, to analyze activator protein 1 (AP-1) transcription factor expression following Rh2 treatment, ten plasmids encoding Renilla luciferase coupled to the transcription factors were transiently transfected into the HepG2 cells and luciferase was detected by the Luciferase Reporter Assay system reagent. The results indicated that ginsenoside Rh2 inhibited HepG2 cell migratory ability. The expression levels of AP-1 transcription factors were increased in HepG2 cells following induction by phorbol 12-myristate 13-acetate, but ginsenoside Rh2 suppressed this induced AP-1 expression. AP-1 transcription factors recruit histone deacetylase (HDAC)4 and affect its transcription, thus, the expression levels of HDAC4 were also analyzed, and these were found to be increased in the Rh2 treatment group. Matrix metalloproteinase 3 (MMP3), a gene downstream of AP-1, was then investigated, and the treatment group expressed reduced levels of MMP3 gene and protein. Therefore, the inhibitory effect of ginsenoside Rh2 on the migratory ability of HepG2 may be presumed to occur by the recruitment of HDAC and the resulting inhibition of AP-1 transcription factors, in order to reduce the expression levels of MMP3 gene and protein.

## Introduction

Hepatocellular carcinoma (HCC) (1–3) is one of the most frequent types of malignancy worldwide and commonly metastasizes to the lung, brain and kidney (4). The incidence of HCC is increasing, particularly in China, due to the high prevalence (one in ten individuals) of hepatitis virus infection, which confers a high risk of HCC (5). Despite advances in diagnosis and treatment, HCC remains the third leading cause of cancer-associated mortality worldwide (6). Surgical resection is the most effective treatment for the majority of HCC patients, but the overall five-year survival rate remains <12% (3). One reason for this is that HCC is commonly detected in other tissues and organs subsequent to surgical removal of the hepatic tumor. Thus, another effective therapy is urgently required to prevent HCC metastasis.

An increasing number of studies have focused on ginseng treatment as an option for HCC. The ginsenoside chemoprevention and anticancer effects are achieved through mechanisms such as DNA damage mitigation, apoptosis induction, proliferation inhibition and positive immunomodulation (7,8). Ginsenoside Rh2 is one of the most widely investigated ginsenosides and exerts potent anticancer activity *in vitro* and *in vivo* (8–11).

Activator protein 1 (AP-1) transcription factors (12) are key downstream targets of the mitogen-activated protein kinase signaling pathway in keratinocytes. AP-1 transcription factors include jun (cjun, junB and junD) and fos (c-fos, FosB, Fra-1 and Fra-2) family members (13,14). These molecules form jun-jun and jun-fos dimers that interact with specific AP-1 transcription factor consensus DNA binding elements in target genes to regulate expression (13). AP-1 transcription factors control keratinocyte proliferation, differentiation and apoptosis, and are important in tumor progression and disease development (15).

An increasing number of transcription factors have been demonstrated to exhibit histone acetyltransferase (HAT) and histone deacetylase (HDAC) activity, and the coexistence of activators with HATs and repressors with HDACs has been frequently identified in transcriptional machinery complexes (16). In addition to modifying chromatin structure, HATs and HDACs associate with additional factors in a number of different cellular processes and function as coordinators and integrators during cell proliferation, differentiation and apoptosis.

Studies have demonstrated that matrix metalloproteinases (MMPs) may be important in HCC development (17,18). MMPs are a family of zinc-dependent proteinases capable of degrading almost all extracellular matrix components, a key event in the majority of malignancies during invasion and metastasis (19,20). Under normal conditions, MMPs are associated with tissue regeneration and wound repair, in addition to reproduction. MMPs may also be involved in carcinogenesis, as previous studies have implicated MMPs in several steps of cancer development, including cancer cell growth, differentiation, apoptosis, invasion and migration; substrates of MMPs include metastatic proteins and growth factor receptors (18,20,22). Overexpression of MMP3 has been observed to be associated with HCC migration (17,23). Ginsenoside Rh2 can inhibit tumor invasion and metastasis, however, the underlying mechanisms remain to be fully elucidated. Thus, the present study was performed in order to further examine the mechanism of ginsenoside Rh2 inhibition of invasion and metastasis in HepG2 liver carcinoma cells.

## Materials and methods

### Cell culture

HepG2 liver carcinoma cells (Bogoo, Shanghai, China) were cryopreserved, then cultured in Dulbecco’s modified Eagle’s medium (DMEM)-F12 containing 10% fetal bovine serum (HyClone, Waltham, MA, USA) at 37°C in an air-5% CO_2_ incubator at constant humidity.

### Antibodies and chemicals

Rh2 (purity 98%) was purchased from National standard network (http://www.gbw114.org/default.asp). Cell Counting kit-8 (CCK-8), fluorescein and liposomes were obtained from Takara Bio, Inc., (Shiga, Japan). A control plasmid (pad-track-tox), which did not encode Renilla luciferase, and the following plasmids encoding the AP-1 transcription factors and Renilla luciferase (luc): p glucocorticoid receptor (GR)-luc, pAP-1-luc, pMYC-luc, p transcription factor (TCF)/lymphoid enhancer-binding factor (LEF)-luc, p retinol binding protein (RBP)/JK-luc, p signal transducer and activator of transcription (STAT)-luc, p hypoxia-inducible factor (HIF)-luc, pE2F/DP1-luc, pSMAD-luc and p nuclear factor of activated T-cells NFAT-luc were provided by Professor Guowei Zuo (Laboratory of Clinical Diagnostics, Chongquing Medical University, Chongqing, China). The primary antibodies used were as follows: histone deacetylase 4 (HDAC4; rabbit monoclonal, 1:1,000) antibody was purchased from Cell Signaling Technology, Inc. (Danvers, MA, USA); AP-1 (rabbit monoclonal, 1:1,000) and MMP3 (rabbit monoclonal, 1:1,000) antibodies were purchased from Sangon Biotech Co., Ltd. (Shanghai, China). The secondary antibodies were as follows: Horseradish peroxidase (HRP)-conjugated goat anti-rabbit immunoglobulin (Ig)G antibody and HRP-conjugated goat anti-mouse IgG antibody were purchased from Beyotime Institute of Biotechnology (Shanghai, China).

### CCK-8 assay

For cell proliferation, a CCK-8 assay was performed (Takara Bio, Inc.). Briefly, 1×10^4^ cells/well were plated in 96-well plates and cultured for the different time periods indicated. At the end of each time period, 20 μl CCK-8 was added to each well and the cells were then incubated at 37°C for 3 h. Subsequently, plates were detected on a spectrophotometric plate reader (Shanghai Precision and Scientific Instrument Co., Ltd., Shanghai, China) at a wavelength of 450 nm.

### Migration assay

To assess cell migratory ability, a Transwell chamber assay (Corning-Costar, Tewksbury, MA, USA) was conducted following the manufacturer’s instructions. This assay examined the ability of the cells to invade through a Matrigel-coated filter. A total of 1×10^4^ HepG2 cells were preteated with Rh2 (80 μmol/l), added to the upper chamber and grown in DMEM-F12 medium on 8.0 μm porous polycarbonate membranes, which were coated with diluted Matrigel basement membrane matrix. The lower chambers were filled with DMEM-F12 medium containing 10% fetal bovine serum. Inside the Transwell chamber, cells were not incubated with Rh2. After 24 h of incubation, any cells remaining on the upper surface of the filter were removed using cotton tips, and the cells that invaded to the lower side of the membrane were fixed with 4% paraformaldehyde for 30 min and then stained with crystal violet for 30 min. The cells in 10 random fields of view at a magnification of ×400 (Shanghai Optical Instrument Factory, Shanghai, China) were counted and the results are expressed as the average number of cells/field of view.

### Immunofluorescence

Sterile glass slides were placed on a six-well plate, then 1×10^7^ cells were seeded. After 24 h, Rh2 (80 μmol/l) was added to the treatment group and DMEM-F12 was added to the control group. The cells were incubated for 24 h and fixed with 4% paraformaldehyde. Cell membranes were ruptured by 0.3% tristone, closed with mountain goat serum (HyClone, Waltham, MA, USA), and the antibodies against AP-1 (1:50) and MMP3 (1:100) were added to the cells, which were incubated overnight. The anti-rabbit secondary fluorescent antibody (dilution 1:500) was subsequently added and the cells were incubated for 1 h, then stained with propidium iodide (Beyotime Institute of Biotechnology, Shanghai, China). Cells were mounted with 50% glycerol and imaged by fluorescent microscopy (Tu Ming Optical Instrument Co., Ltd.).

### Transient transfection and luciferase activity assays

HepG2 cells were transiently transfected in triplicate using effectene transfection reagent (Takara Bio, Inc.). The plasmid encoding Renilla luciferase (pad-track-tox) served as a control. The plasmids encoding AP-1 transcription factors coupled with Renilla luciferase, namely pGR-luc, pAP-1-luc, pMYC-luc, pTCF/LEF-luc, pRBP/JK-luc, pSTAT-luc, pHIF-luc, pE2F/DP1-luc, pSMAD-luc and pNFAT-luc, were transfected for normalization of transfection efficiency, unless otherwise indicated. The cells were trypsinized and evenly distributed into the wells of a six-well plate prior to designation of the treatment conditions. At 24 h following transfection, the cells were treated with 80 μM Rh2. The cell supernatants were assayed for Renilla luciferase activities using the Luciferase Reporter Assay system reagent (Takara Bio, Inc.), according to the manufacturer’s instructions.

### Western blot analysis

The protein content of cell extracts was determined by a Bradford assay (Bio-Rad, Hercules, CA, USA). A total of 20–30 μg protein was electrophoresed by 10–15% SDS-PAGE and transferred to polyvinylidene difluoride membranes. The membranes were blocked and incubated with the primary antibodies at the appropriate concentrations, and subsequently incubated with HRP-conjugated goat anti-rabbit or goat anti-mouse IgGs (1:2,000; Beyotime Institute of Biotechnology). Labeled bands were detected by the Immun-Star™ HRP Chemiluminescent kit (Bio-Rad), and images were captured and the intensity of the bands was quantified by the VersaDoc™ image system (Bio-Rad, Regents Park, Australia).

### Reverse transcription polymerase chain reaction (RT-PCR) analysis

RT-PCR was performed using the ABI Prism 7700 sequence detection system (PE Applied Biosystems; Foster City, CA, USA). Total RNA was extracted using TRIzol Reagent (Invitrogen Life Technologies, Carlsbad, CA, USA). RT-PCR was conducted using Moloney murine reverse transcriptase and Oligos (T), and the resulting cDNA products were used as templates for PCR assays. A volume of 25 μl mixture was used for the subsequent PCR reaction. The fold-change in gene expression levels was determined using the 2^−ΔΔCT^ method with beta-actin serving as an endogenous control. The primer sequences were as follows: AP-1 forward, GCAAACCTCAGCAACTTCAACC and reverse, GCATCTCGGGCACTGTCTGA; MMP3 forward, TAATGGAGATGCCCACTTTGATG and reverse, GAGTGAAAGAGACCCAGGGAGTG. Following incubation at 5°C for 2 min followed by 95°C for 10 min, the reaction was performed for 40 cycles of the following: 95°C for 15 sec and 60°C for 1 min.

### Statistical analysis

Statistical significance of the differences between control and treated samples was calculated using Student’s t-test (SSPS 17.0; SPSS, Inc., Chicago, IL, USA). P<0.05 was considered to indicate a statistically significant difference. All the experiments were repeated at least three times, each time with three or more independent observations.

## Results

### HepG2 cell migration is inhibited by Rh2

**Figure 1:**
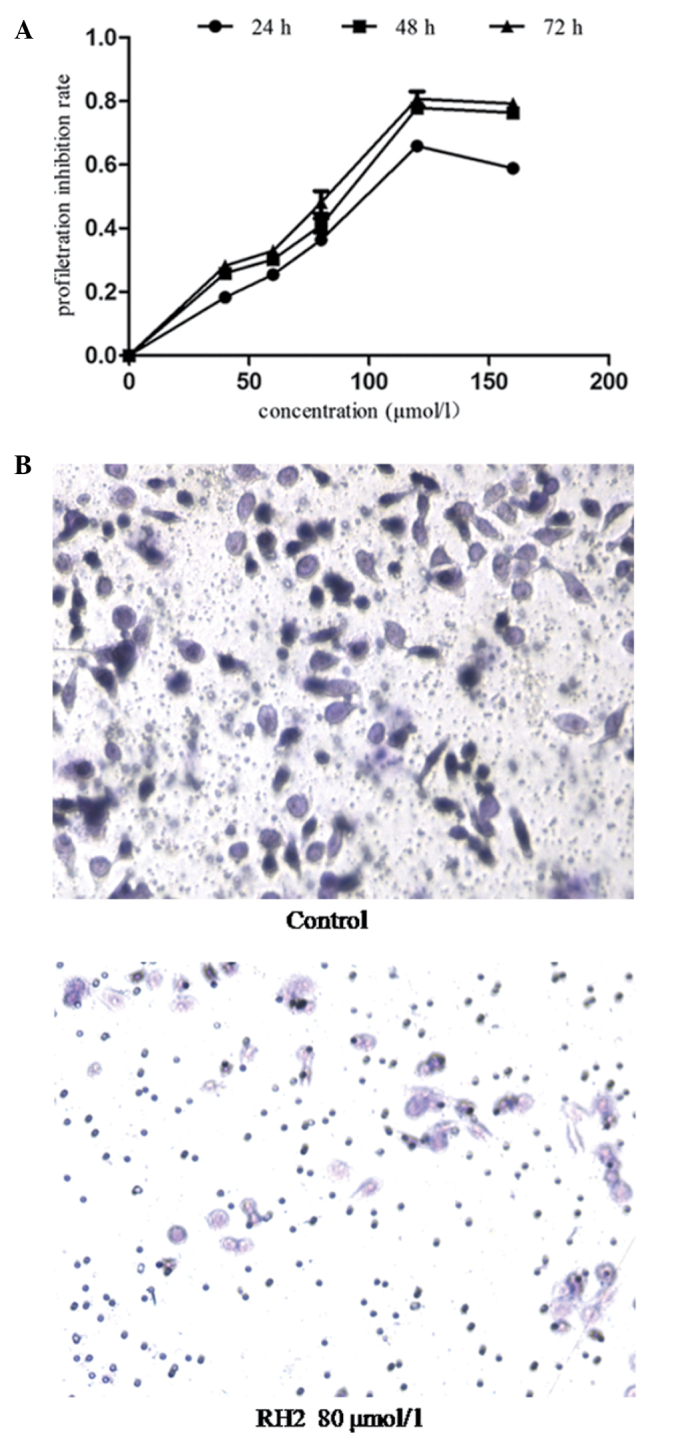
Growth and migration of HepG2 liver carcinoma cells. (A) Cells were incubated with ginsenoside Rh2 (10–160 μ/mol) for 24, 48 and 72 h, and then assessed by cell counting kit-8 assay. Cell growth occurred in a dose-dependent manner. Each point indicates mean ± standard deviation (n=6). (B) Transwell assay of HepG2 cells incubated with Rh2 (80 μmol/l) or pure medium (control). Migrated cells were stained with crystal violet, and cell morphology and quantity were observed with an inverted microscope (magnification ×400). Results shown are representative of at least three independent experiments. ^*^P<0.05 vs. control.

Previous studies have observed that ginsenoside Rh2 induces apoptosis, inhibits cell proliferation, improves immunomodulation and perturbs cycle arrest, which provides a potent anticancer effect (7). In the present study, CCK8 assay was used to confirm the anticancer effect of ginsenoside Rh2. The results indicated that Rh2 inhibited the survival of HepG2 cells at various concentrations ranging from 10–160 μmol/l. Compared with the untreated group, cell proliferation was significantly reduced by Rh2 (10–160 μmol/l) in the treated group (P<0.01). In addition, HepG2 cell growth inhibition by Rh2 was dose- and time-dependent (Fig. 1A). Therefore, the effect of ginsenoside Rh2 on cancer growth was significant. In order to examine whether ginsenoside Rh2 inhibits tumor migratory ability, the Transwell assay was used. The results revealed that, compared with the treatment group, a significantly greater number of HepG2 cells in the control group remained in the chamber. Thus, Rh2 inhibited HepG2 cell migratory ability (P<0.05; Fig. 1B).

### AP-1 transcription factors are inhibited by Rh2

**Figure 2:**
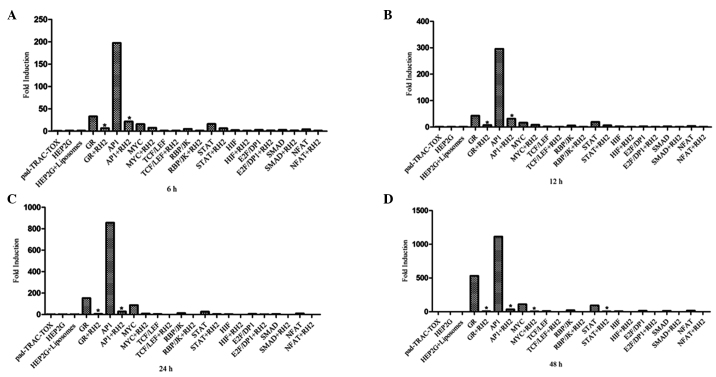
AP-1 transcription factors in HepG2 liver carcinoma cells were identified, and the mRNA expression levels following various durations of ginsenoside Rh2 treatment were analyzed. (A–D) Cells were trypsinized and evenly distributed in the wells of a six-well plate prior to designation of treatment condition. After 4 h, the cells were either transfected with plasmid (pad-track-tox) as a positive control, left untreated as a negative control, transfected with liposomes as a false positive or transfected with the following plasmids encoding AP-1 transcription factors and Renilla luciferase: pGR-luc, pAP-1-luc, pMYC-luc, pTCF/LEF-luc, pRBP/JK-luc, pSTAT-luc, pHIF-luc, pE2F/DP1-luc, pSMAD-luc or pNFAT-luc, designated as the treated groups. The treated groups were then divided two subgroups, designated group A: GR, AP-1, MYC, TCF, RBP/JK, STAT, HIF, E2F/DP1, SMAD and NFAT; and B: GR+Rh2, AP-1+Rh2, MYC+Rh2, TCF+Rh2, RBP/JK+Rh2, STAT+Rh2, HIF+Rh2, E2F/DP1+Rh2, SMAD+Rh2 and NFAT+Rh2. The B group was then treated with 80 μM ginsenoside Rh2. After 6, 12, 24 and 48 h, the cell supernatants were assayed for Renilla luciferase activities using the Luciferase Reporter Assay system reagent and assayed according to the manufacturer’s instructions. Results shown are representative of at least three independent experiments.^*^P<0.05 vs. control. AP-1, activator protein 1; luc, luciferase; GR, glucocorticoid receptor; TCF, transcription factor; LEF, lymphoid enhancer-binding factor; STAT, signal transducer and activator of transcription; HIF, hypoxia-inducible factor; RBP, retinol binding protein; NFAT; nuclear factor of activated T-cells.

The aforementioned results suggested that Rh2 inhibited HepG2 cell migratory ability; thus, the mechanism of action was investigated. A total of 10 plasmids encoding Renilla luciferase were transiently transfected into cells in group A, serving as the control group, and group B, to which 80 μM ginsenoside Rh2 was added. After 6, 12, 24 and 48 h, the cell supernatants were assayed for Renilla luciferase activity using Luciferase Reporter Assay system reagent to analyze AP-1 transcription factor mRNA expression levels. The results indicated that the pGR-luc and pAP-1-luc activities in group A were higher than in group B, particularly the pAP-1-luc activity, which was significantly increased in group A (P<0.05; Fig. 2).

**Figure 3:**
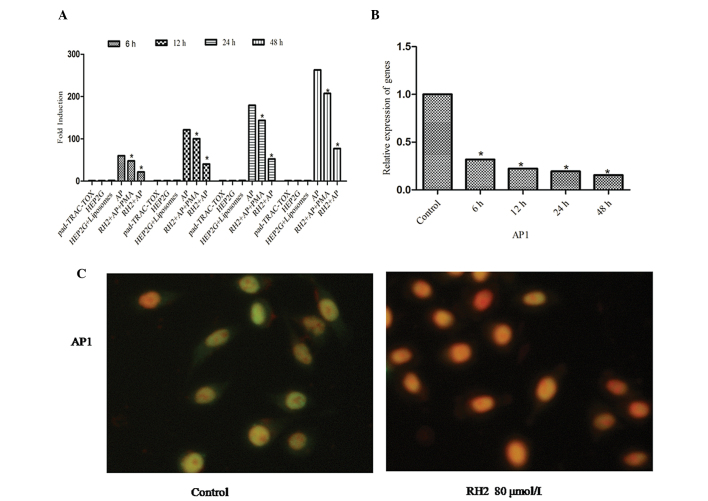
AP-1 transcription factors were inhibited by ginsenoside Rh2 in HepG2 liver carcinoma cells. (A) Cells were transfected with either plasmid (pad-track-tox) as a positive control, left untreated as a negative control, transfected with liposomes as a false positive or transfected with plasmid encoding Renilla luciferase (pAP-1-luc) as the treated group. The treated group was divided into three subgroups, designated A1 (AP-1), B1 (AP-1+Rh2+PMA) and C1 (AP-1+Rh2). The B1 group was treated with 80 μM ginsenoside Rh2 and 30 μM PMA. The C1 group was treated with 80 μM Rh2 only. (B) HepG2 cells were incubated for 6, 12, 24 and 48 h with Rh2 (80 μM). The expression levels of AP-1 gene were measured by reverse transcription polymerase chain reaction. (C) Fluorescence microscopy images of AP-1 expression in isolated single cells (magnification, ×400). Results shown are representative of at least three independent experiments.^*^P<0.05 vs. control. AP-1, activator protein 1; PMA, phorbol 12-myristate 13-acetate.

Mittelstadt and Patel (13) reported that phorbol 12-myristate 13-acetate (PMA) activated AP-1 transcription factor expression; therefore, in the present study, PMA was employed to induce AP-1 transcriptor factor mRNA expression. In order to demonstrate that Rh2 inhibits induced AP-1 transcription factor expression, pGR-luc plasmids were once again transiently transfected into HepG2 cells. The results revealed that Rh2 inhibited AP-1 transcription factor mRNA expression, although the luciferase activity in the AP-1 + Rh2 + PMA group was higher than that in the AP-1 + Rh2 group. This again demonstrated that AP-1 transcription factor mRNA expression was inhibited by Rh2 (Fig. 3A). The expression levels of the AP-1 gene itself were also analyzed; RT-PCR results indicated that Rh2 inhibited AP-1 gene expression (Fig. 3B). Fluorescence microscopy was used to detect any subsequent changes in AP-1 protein expression, and Rh2 was shown to also inhibit the expression of AP-1 protein (Fig. 3C).

### Increased HDAC4 expression levels are induced by Rh2

**Figure 4:**
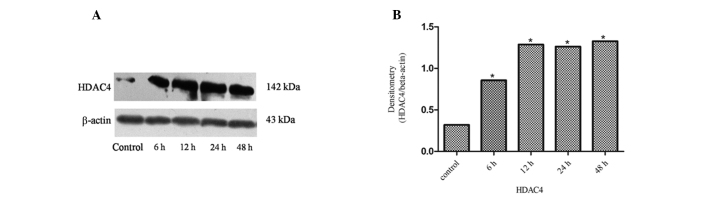
Increased HDAC4 expression levels induced by ginsenoside Rh2 in HepG2 liver carcinoma cells. (A and B) Cells were incubated 6, 12, 24 and 48 h with Rh2 (80 μM). HDAC4 expression levels were determined by western blotting; beta-actin served as a protein loading control. Results shown are representative of at least three independent experiments.^*^P<0.05 vs. control. HDAC4, histone deacetylase 4.

HDAC4 is recruited by AP-1 to inhibit MMP3 protein expression (13). HDACs are important in the epigenetic regulation of gene expression through catalyzing the removal of acetyl groups, stimulating chromatin condensation and promoting transcriptional repression. Mittelstadt and Patel (13) reported that the AP-1 family member c-jun interacts with HDAC3 to repress transcription of target promoters. In the present study, ginsenoside Rh2 was observed to increase the expression levels of HDAC4 (Fig. 4). Therefore, recruitment of HDAC4 to the proximal AP-1 binding site was presumed to result in repression of AP-1 transcription factors.

**Figure 5:**
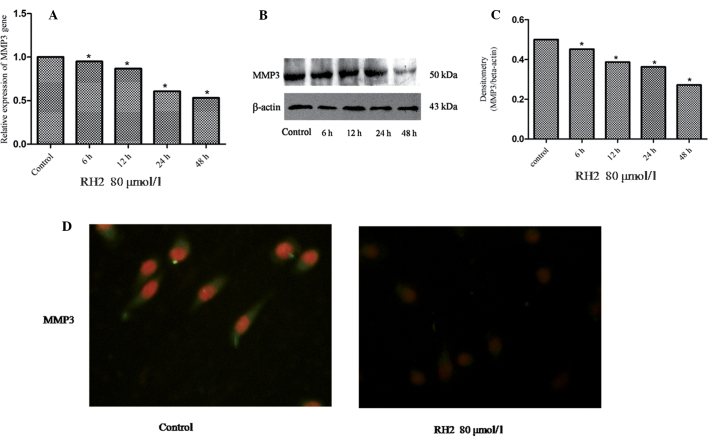
MMP3 mRNA and protein expression were inhibited by ginsenoside Rh2. HepG2 liver carcinoma cells were incubated for 6, 12, 24 and 48 h with Rh2 (80 μM). (A) The expression levels of the MMP3 gene were measured by reverse transcription polymerase chain reaction. (B and C) The expression levels of MMP3 protein were determined by western blotting; beta-actin served as a protein loading control. (D) Fluorescence microscopy images of MMP3 expression in isolated single cells. Results shown are representative of at least three independent experiments (magnification, ×400).^*^P<0.05 vs. control. MMP, matrix metalloproteinase.

The MMP3 gene is downstream of the AP-1 transcription factors. The expression levels of MMP3 mRNA were examined by RT-PCR and the results indicated that Rh2 inhibited MMP3 gene expression (Fig. 5A). MMP3 protein expression levels were detected through western blot analysis; the results indicated that Rh2 inhibited the expression of MMP3 protein (Fig. 5B and C). This result was verified by fluorescence microscopy findings (Fig. 5D). AP-1 usually functions as a transcription factor. Under non-induced conditions, general transcription factors occupy the MMP-3 promoter site. Following induction by Rh2, HDAC4 was recruited to the MMP-3 promoter site. Consequently, reduced local histone acetylation was identified following Rh2 exposure, which conferred a more tightly packed chromatin structure and may have resulted in reduced MMP-3 transcription. The results describe a mechanism whereby inhibition of transcription factor AP-1 downregulated MMP-3 gene transcription.

## Discussion

*Panax ginseng* has been used to cure diseases in Chinese Traditional Medicine for thousands of years and has also been employed in recent years for adjuvant therapy in various types of cancer (9). Rh2 has been found to exert a marked effect on inducing apoptosis in pancreatic cancer cells, hepatoma cells and A549 lung cancer cells (9,11,24,25). Thus, the effect of ginsenoside Rh2 on cancer is evident, but its ability to inhibit tumor migration has not been elucidated. The present study was undertaken to gain insight into the molecular mechanisms of ginsenoside Rh2 inhibition of tumor migration in HepG2 liver carcinoma cells. In order to investigate this, Transwell chambers were used to examine cell migratory ability. The data revealed that HepG2 cell migratory ability was markedly inhibited by Rh2 (Fig. 1B).

The luciferase reporter gene assay is an important means to detect the expression of transcription factors and specific transcription factor binding target promoter sequences (26,27). In order to investigate the transcription factors active in HepG2 cells, plasmids encoding Renilla luciferase, namely pGR-luc, pAP-1-luc, pMYC-luc, pTCF/LEF-luc, pRBP/JK-luc, pSTAT-luc, pHIF-luc, pE2F/DP1-luc, pSMAD-luc and pNFAT-luc, were transfected into the cells. The data indicated that pAP-1-luc activities were significantly increased in the HepG2 cells, indicating that the AP-1 transcription factors were active. AP-1 transcription factors mainly consist of Fos and Jun family proteins that form homodimers or heterodimers, which bind DNA through conserved bZIP domains. Recent studies have focused on AP-1 transcription factor dynamics, and how these modify gene expression (28). Increasing or reducing AP-1 transcription factors enhances or inhibits gene transcription. Of note, in the present study, Rh2 inhibited pAP-1-luc luciferase activity, which suggested that Rh2 remodels AP-1 transcription factors. Miotto *et al* (29) reported that the interactions among AP-1 and partner molecules modified and remodeled chromatin by recruiting DRpd3/HDAC1 to reverse histone acetylation. Other reports on the stimulation of Fos or Jun activities by CREB-binding protein suggested that the recruitment of HAT coactivator complexes at target promoters mediates nucleosome acetylation and stimulates transcription (29). In the present study, western blotting was used to detect HDAC4 expression levels and of note, Rh2 was observed to affect these expression levels. Therefore, it was hypothesized that AP-1 recruits HDAC4 and affects its transcription simultaneously (30). The function of HDAC4 in the control of the c-Jun N-terminal kinase signaling pathway appears specific, which appears to be the mode of action of HDAC4 regulating AP-1 (31).

MMPs are a extensive family of zinc-dependent proteinases that are key in extracellular matrix degradation; MMPs alter cell motility by exposing cryptic matrix signals (32). Tumor cells are hypothesized to secrete and/or induce host cells to produce these matrix-degrading enzymes. The majority of studies suggest that increased expression levels of MMP-2, -3 and -9 proteins in tumors correlate with poorer prognosis (33). MMPs are regulated at the transcription level by a variety of cytokines, chemokines and growth factors. Butticè *et al* reported that MMP1 and MMP3 genes are regulated by the Ets and Fos/Jun transcription factors/oncoprotein families (34). In the present study, the MMP3 gene, downstream of AP-1, exhibited reduced expression levels, concurrent with simultaneously reduced AP-1 gene expression levels (Fig. 5). Numerous inducible MMP genes, including those of MMP1, MMP3, MMP7, MMP9, MMP10, MMP12 and MMP13, contain promoter-proximal AP-1 sites, which are key features of inducibility. Therefore, the modification of AP-1 may be presumed to affect MMP3 gene expression.

In conclusion, Ginsenoside Rh2 markedly inhibited the migration ability of HepG2 cells; the present study was undertaken to gain insight into the molecular mechanisms of this effect. Ginsenoside Rh2 was presumed to affect the migratory ability of HepG2 cells by recruiting HDAC, thus inhibiting AP-1 transcription factors, which reduced the expression levels of MMP3 mRNA and protein.
